# USP1 Promotes GC Metastasis via Stabilizing ID2

**DOI:** 10.1155/2021/3771990

**Published:** 2021-11-27

**Authors:** Nuoya Li, Lei Wu, Xingye Zuo, Huilong Luo, Yanling Sheng, Jinlong Yan

**Affiliations:** ^1^Department of General Surgery, Second Affiliated Hospital of Nanchang University, Nanchang, 330006 Jiangxi Province, China; ^2^Department of General Surgery, Yongxin County People's Hospital, Ji'an, 343000 Jiangxi Province, China; ^3^Department of Ultrasound, The Affiliated Hospital of Jiangxi University of Traditional Chinese Medicine, Nanchang, 330006 Jiangxi Province, China

## Abstract

Gastric cancer (GC) is one of the most common malignant tumors all over the world. And recurrence and metastasis are still the main causes of low survival rate for advanced GC. USP1 has been shown overexpressed in multiple cancers, which indicate its important biomarker in tumorigenesis and development. Our study is aimed at defining the exact role of USP1 on GC metastasis and the underlying mechanism. USP1 was firstly found overexpressed in GC tissues and relatively high-expression levels conferred poor survival rates. Then, real-time cellular analysis (RTCA) showed that USP1 knockdown inhibited GC metastasis both in vitro and in vivo. Mechanically, we demonstrated that USP1 promoted GC metastasis via upregulating ID2 expression and further confirmed that USP1 stabilized ID2 expression through deubiquitinating ID2 in GC. In conclusion, our study showed that USP1 promoted GC metastasis via stabilizing ID2 expression, which provides a potential biomarker and therapy target for GC.

## 1. Introduction

Gastric cancer (GC) is one of the most common malignant tumors all over the world, with estimated 951,600 new cases and 723,100 deaths occurring in 2012 [1]. Although most countries have shown declining trend in incidence and mortality rates of GC over the past decades [2, 3], the absolute incidence is increasing owing to the growth and aging of population worldwide [4]. Endoscopic submucosal dissection or surgical resection of stomach is still the best choice for early GC, but for advanced patients, even with maximal treatments, the overall survival rates for 5 years are still low owing to recurrence and metastasis [5]. Molecular target treatment has shown its advantages in improving the prognosis of advanced GC patients in recent years [6]. Thus, it is necessary to further explore the molecular mechanisms of GC metastasis and search for new treatment strategies for GC.

Ubiquitination is an important posttranscriptional modification (PTM) which participate in a number of cellular processes, such as protein degradation, gene expression, and DNA repair [7]. Similar to other posttranscriptional modifications, ubiquitin modification is also a reversible process. Deubiquitinases (DUBs) can exert deubiquitination effect by hydrolyzing the isopeptide (or peptide) bond between ubiquitin or ubiquitin-like proteins and target proteins, so as to inhibit protein degradation and rescue their initial functions [8, 9]. Based on the Ub-protease domains in the human genome, DUBs are divided into 5 subclasses, of which ubiquitin-specific protease (USP) is the largest subclass [10]. USP has been indicated its regulatory effects on the development and progression of cancer [11–13]. As a member of USPs, USP1 plays important role in DNA repair through deubiquitinating Fanconi anemia complementation group I (FANCI), Fanconi anemia group D2 (FANCD2), and proliferating cell nuclear antigen (PCNA) [10, 14, 15]. Thus, USP1 is associated with multiple diseases including cancer. Previous studies have shown that USP1 is overexpressed in multiple cancers, such as osteosarcoma, multiple myeloma, glioblastoma, and non-small-cell lung cancer (NSCLC) [16–19]. But in the research of Zhiqiang et al., low expression of USP1 was found in non-small-cell lung cancer tissues and overexpressing USP1 inhibited lung cancer cell proliferation [20]. Thus, it is controversial whether USP1 acts as an oncogene or tumor suppressor in the tumorigenesis and progression. In our study, we aimed to define the exact role of USP1 in GC metastasis and further to explore the underlying mechanism.

DNA-binding protein inhibitor ID-2 is a protein that in humans is encoded by the ID2 gene. ID2 may play a role in negatively regulating cell differentiation. Recently, its roles in cancers have been increasingly studied [21]. A research from Italian researchers states that ID2 protein has a relevant role in the development and resistance to therapies of glioblastoma, the most aggressive of brain cancers [22]. Another study found an inhibitor of DNA binding 2 (ID2) as a novel molecule involved in the regulation of invasion and LNM of HNSCC and further verified its functional role. Overexpression of ID2-induced invasion and LNM of HNSCC cells was observed in vitro and in vivo. By contrast, knockdown of the ID2 gene diminished invasion and LNM of HNSCC cells [23]. In addition, targeting ID2 could significantly inhibit the proliferation of colorectal cancer cells in mice [24]. However, the regulation of ID2 in gastric cancer remains unclear.

## 2. Materials and Methods

### 2.1. Patients and Samples

GC specimens were collected from 188 patients who underwent GC resection at the Second Affiliated Hospital of Nanchang University between January 2010 and May 2014. Informed consent was obtained from each patient, and the study protocol was approved by the Ethics Committee of the Second Affiliated Hospital of Nanchang University.

### 2.2. Cell Culture

The human GC cell lines BGC-823 and MGC-803 and human gastric mucosal cell line GES-1 were purchased from the Shanghai Cell Bank, Type Culture Collection Committee of Chinese Academy of Science (Shanghai, China). All cell lines were authenticated using short tandem repeat profiling by the Cell Bank. The cells were cultured in DMEM or MEM (Gibco, Grand Island, NY, USA) supplemented with 10% fetal bovine serum (Gibco, Grand Island, NY, USA) at 37°C and under 5% CO2.

### 2.3. Plasmids and Reagents

**Figure 1:**
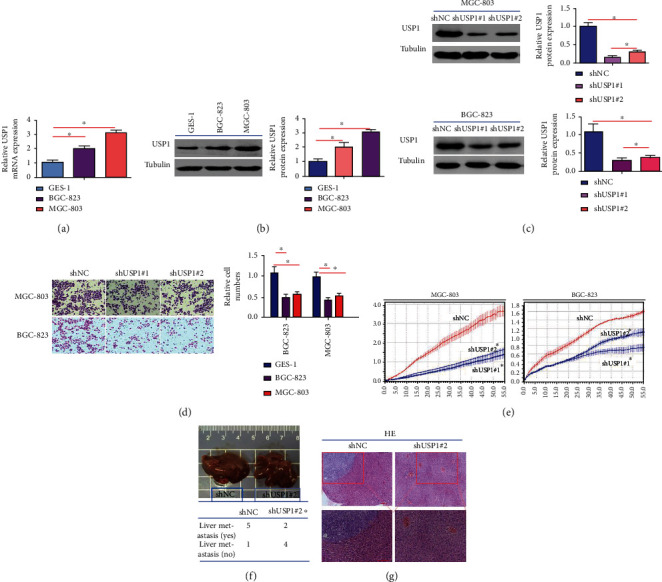
Stable knockdown of USP1 inhibits GC metastasis both in vitro and in vivo. (a, b) mRNA and protein expression levels of USP1 in normal gastric mucosa cells and two human GC cells (GES-1, BGC-823, and MGC-803) were detected by RT-qPCR and Western blot; (c) the efficacy of shRNA1 and shRNA2 mediated knockdown of USP1 was detected by Western blot; (d, e) in vitro experiments, MGC-803 and BGC-823 cells were transfected with shUSP1#1 or shUSP1#2; the real-time cellular analysis (RTCA) were used to evaluate cell migration; (f) in vivo experiments, MGC-803 cells transfected with shUSP1 or shNC were injected into the tail veins of nude mice; the representative images of HE staining of liver tissues with metastatic cells and the number of mice with liver metastasis were shown (^∗∗^*p* < 0.01; ^∗^*p* < 0.05).

Based on the USP1 (NM_001017415.2) and ID2 (NM_002166.5) sequences, two shRNAs were designed using the siRNA Target Finder (InvivoGen). The target sites of shRNAs are detailed in Supplementary Table S1. The interference effects were confirmed by Western blotting (Figure 1(c)). The shUSP1 and shID2 construct that produced the most significant knockdown effect was used to transduce GC cells. Stably transfected GC cells were selected based on resistance to hygromycin (600 *μ*g/ml) (Invitrogen, Carlsbad, CA, USA), and GC cells transfected with a negative control vector (shNC) were included as a control. pCMV-flag-USP1, pCMV-HA-Ub, and pCMV-his-ID2-expressing GC cells were selected using G418 (700 *μ*g/ml) (Invitrogen), and an empty vector was used as the negative control. After four weeks of selection, individual colonies were isolated and expanded. All primers are listed in Supplementary Table S1.

The following antibodies and reagents/kits were used: USP1, ID2, flag, his, HA, and Tubulin (Proteintech, Chicago, IL, USA); ubiquitin (Ub) (Nova Biomedical, MA, USA); protein A/G PLUS-agarose (Santa Cruz, CA, USA); DMEM, fetal bovine serum, and Lipofectamine® 3000 (Invitrogen); total protein extraction kit (Applygen, Beijing, China); BCA protein quantitation kit (Beyotime, Jiangsu, China); CHX [25] and PS-341 [26]; and MG132 [27] (Selleck, Houston, USA).

### 2.4. Immunohistochemistry (IHC)

188 GC and adjacent tissue (nontumor) sections were treated with xylene and graded alcohol and then subjected to antigen retrieval in 0.01 M citrate buffer. Hydrogen peroxide was used for blockage. The sections were incubated with goat serum for 30 min and then with anti-USP1 polyclonal antibodies (Proteintech, Chicago, IL, USA, 1 : 100 dilution) overnight at 4°C. A 2-step immunohistochemical method (catalog no.: PV-9000; ZSGB-BIO Co., Ltd., Beijing, China) was adopted for immunostaining. The staining intensity and percentage of positive cells were scored semiquantitatively by 3 pathologists who were blind to the clinical parameters.

### 2.5. Real-Time Quantitative Polymerase Chain Reaction (qRT-PCR), Western Blot Analysis, and Coimmunoprecipitation (Co-IP)

qRT-PCR, Western blotting, and Co-IP were performed as previously described [28, 30]. The primers for q-PCR were included in Supplementary Table 1.

### 2.6. Transwell Assay

To determine cell invasion and migration, the cells were seeded on the top chamber of a Transwell (Corning, USA) with serum free medium, while complete medium containing 10% FBS were added in the lower chamber. After 24-hour incubation, the migrated cells were fixed with 4% PFA and then stained with 0.1% crystal violet (Sigma, USA) for 20 minutes. The results were photographed under an invert microscope. We also detected the cell invasion with the same protocol, but the membranes were coated with Matrigel (Stemcell, USA).

### 2.7. Real-Time Proliferation Assay (RTCA)

Real-Time Cell Kinetic Analyzer xCELLigence RTCA (ACEA Biosciences) was used to monitor cell invasion and migration. Data were analyzed using the RTCA Control Unit and the preinstalled RTCA software. For real-time proliferation assay, CIA-plate 16 was used. Cells were seeded directly onto the CIA-plate. Changes in baseline impedance resulting from an increase in cell number were monitored by gold microelectrodes located at the bottom of CIA-plate 16. The proportional changes in impedance were recorded continuously and expressed as cell index. Changes in cell index over time were monitored continuously.

### 2.8. Establishment of Liver Metastasis Model of Gastric Cancer in Nude Mice

MGC-803 cells were transfected with USP1 interference empty plasmid (shNC) and USP1 interference plasmid (shUSP1) before injection. The concentration was 1 × 0.2 ml of 10^7^ human gastric cancer cell MGC-803 suspension inoculated under the splenic capsule of nude mice. The mice were killed at the 4th week after operation. The liver metastasis was observed by HE staining. There were 6 nude mice in the two groups. Animal: all the animal work was approved by the Ethics Committee for Animal Experiments of the Second Affiliated Hospital of Nanchang University.

### 2.9. Statistical Analysis

Data were analyzed using GraphPad Prism v7.0 (GraphPad Software Inc., USA). Survival curves were generated using the Kaplan-Meier method. Differences between groups were analyzed using Student's *t*-test when comparing two groups or by one-way analysis of variance (ANOVA) when comparing more than two groups. *p* < 0.05 or 0.01 was considered significant.

## 3. Results

### 3.1. USP1 Is Overexpressed in in GC Tissues, and Relatively High Expression Levels of USP1 Correlate with Poor Survival

**Figure 2:**
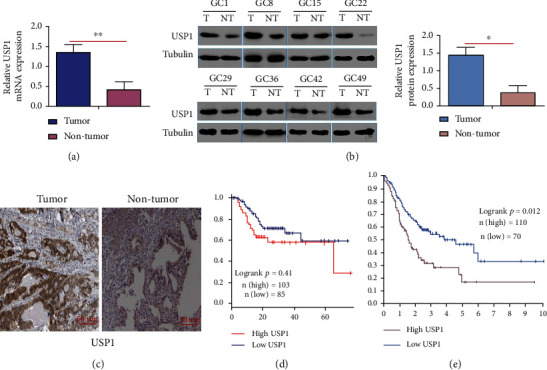
USP1 is overexpressed in in GC tissues and relatively high expression levels of USP1 correlates with poor survival. (a, b) mRNA and protein expression levels of USP1 were detected by RT-qPCR and Western blot; *β*-Tubulin was used as an internal control; (c) the representative images of USP1 staining in GC tissues and adjacent normal tissues were shown by immunohistochemistry (IHC); (d) based on the USP1 expression, the overall survival of GC patients were analyzed by Kaplan-Meier curve; (e) data from the TCGA database was further used to analyze the overall survival rate of GC patients (^∗∗^*p* < 0.01; ^∗^*p* < 0.05).

RT-qPCR and Western blot results further showed that the mRNA levels and protein levels of USP1 in GC tissues were higher than that in adjacent normal tissues (Figures 2(a) and 2(b)). In order to compare the expression levels USP1 on GC tissues and adjacent normal tissues from 116 patients, immunohistochemistry (IHC) was performed. The results showed that more intensive USP1 staining was found in GC tissues than that in adjacent normal tissues (Figure 2(c)). Kaplan-Meier analysis indicated that patients with relatively high expression levels had lower survival rates than those with low expression levels (Figure 2(d)), which was further confirmed by data from TCCA database (Figure 2(e)).

### 3.2. Stable USP1 Knockdown Inhibits GC Metastasis Both In Vitro and In Vivo

In order to define the exact role of USP1 on GC, USP1 knockdown was performed both in vitro and in vivo. USP1 was highly expressed in all human GC cells compared with normal control (Figures 1(a) and 1(b)). MGC-803 and BGC-823 cells which had relatively higher expression levels of USP1 were transfected with shUSP#1 or shUSP#2, and the efficacy of USP1 knockdown was detected by Western blot (Figure 1(c)). As shown in Figure 1(d), USP1 knockdown significantly inhibited cell migration in MGC-803 and BGC-823 cells in Transwell experiments (Figure 1(d)). Real-time dynamic curves further showed that less number of migrated cells were observed when transfected with shUSP1 (Figure 1(e)). In order to evaluate the effects of USP1 on distant metastasis in vivo, MGC-803 cells transfected with shUSP1 or shNC were inoculated under the splenic capsule of nude mice. USP1 knockdown significantly decreased the number of mice with liver metastasis (Figures 1(f) and 1(g)). Overall, these results demonstrate that USP1 knockdown inhibits GC migration both in vitro and in vivo.

### 3.3. USP1 Promotes GC Metastasis by Upregulating ID2 Expression

**Figure 3:**
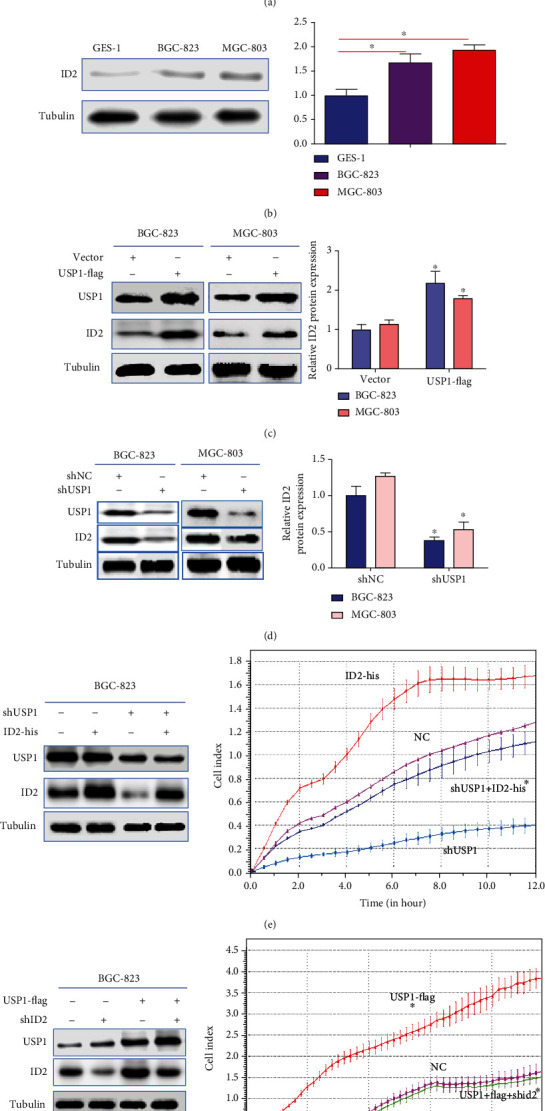
USP1 promotes GC metastasis by upregulating ID2 expression. (a, b) The mRNA and protein levels of ID2 in normal gastric mucosa cells and two human GC cells were detected by RT-qPCR and Western blot; (c) MGC-803 and BGC-823 cells were transfected with flag-vector or USP1-flag; the protein expression levels of USP1 and ID2 were detected by Western blot; (d) MGC-803 and BGC-823 cells were transfected with shNC or shUSP1; the protein expression levels of USP1 and ID2 were detected by Western blot; (e) BGC-823 cells were transfected with shUSP11 and/or his- ID2, the protein expression levels of USP1 and ID2 were detected by Western blot, and the effects of cell migration were detected by RTCA. (f) BGC-823 cells were transfected with flag-USP1 and/or shID2, the protein expression levels of USP1 and ID2 were detected by Western blot, and the effects of cell migration were detected by RTCA (^∗∗^*p* < 0.01; ^∗^*p* < 0.05).

Previous studies have shown that inhibitor of DNA binding-2 (ID2) is overexpressed in multiple cancers including gastric cancer [28–31]. To define the correlation between USP1 and ID2 in GC, the expression levels of USP1 and ID2 were firstly detected by RT-qPCR and Western blot (Figures 3(a) and 3(b)). Conversely, flag-USP1-mediated USP1 overexpression increased the expression levels of ID2 (Figure 3(c)). Then, BGC-823 and MGC-803 cells were transfected with shUSP1; Western blot results showed that USP1 knockdown significantly decreased the expression levels of ID2 (Figure 3(d)). Thus, we speculated that the mechanism by which USP1-promoted GC migration was associated with upregulating ID2 expression. To confirm our hypothesis, BGC-823 were transfected with shUSP1 and/or his-ID2; Western blot results showed that USP1 knockdown significantly decreased the expression levels of ID2 and reduced the number of migrated cells, which was rescued by ID2 overexpression (Figure 3(e)). Conversely, USP1 overexpression significantly increased the expression levels of ID2 and increased the number of migrated cells, which was inhibited by ID2 knockdown (Figure 3(f)). Overall, these results demonstrate that USP1 promotes GC metastasis by upregulating ID2 expression.

### 3.4. The Degradation of ID2 Depends on Ub-Proteasome Way in GC

**Figure 4:**
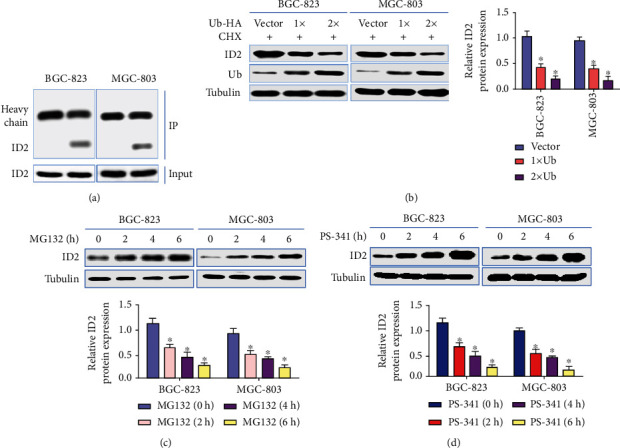
ID2 degradation depends on Ub-proteasome way. (a) Coimmunoprecipitation assay was used to detected ubiquitinated ID2 in MGC-803 and BGC-823 cells; (b) MGC-803 and BGC-823 cells were transfected with increasing amounts of HA-Ub, then exposed to CHX; the protein expression levels of ID2 and Ub were detected by Western blot; (c, d), GC-803 and BGC-823 cells were exposed to proteasome inhibitors-MG132 and PS-341, respectively; the protein expression levels of ID2 were detected by Western blot at indicated time after treatment (^∗∗^*p* < 0.01; ^∗^*p* < 0.05).

Previous study has shown that ID2 is normally polyubiquitinated and can be rapidly degraded by the Ub-proteasome pathway [31]. For further confirming the ubiquitination and degradation of ID2 in GC, Co-IP assay between endogenous ID2 and Ub was firstly performed in MGC-803 and BGC-823 cells. As shown in Figure 4(a), ID2 could be detected when Ub was immunoprecipitated. Then, MGC-803 and BGC-823 cells were transfected with increasing amount of HA-Ub and exposed to cycloheximide(CHX). CHX can inhibit protein synthesis by interfering the translation of mRNA [25]. Western blot results showed that the expression levels of ID2 were decreased with increasing expression levels of Ub (Figure 4(b)). In order to define the effect of proteasome on the degradation of ID2, proteasome inhibitors (MG132 and PS-341) [26, 27] were added to MGC-803 and BGC-823 cells; Western blot was used to detect the expression levels of ID2. As shown in Figures 4(c) and 4(d), endogenous ID2 was accumulated with increasing time. Overall, these results demonstrate that the degradation of ID2 depends on Ub-proteasome way in GC.

### 3.5. USP1 Stabilizes ID2 Expression through Deubiquitinating ID2 in GC

**Figure 5:**
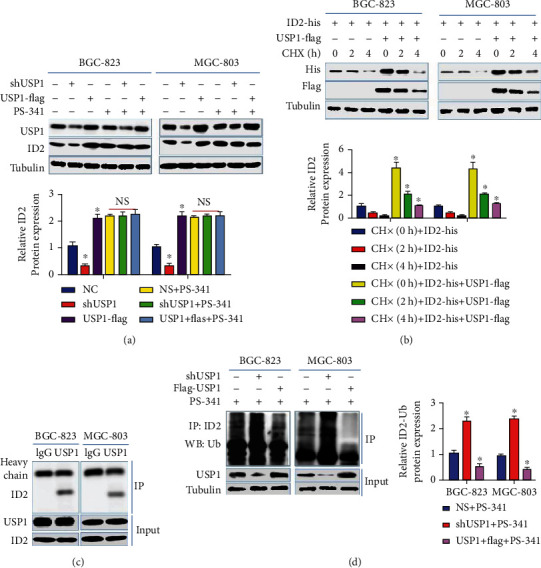
USP1 stabilizes ID2 expression through deubiquitinating ID2. (a) MGC-803 and BGC-823 cells were transfected with shUSP1 or flag-USP1, then exposed to PS-341 or not; the protein expression levels of USP1 and ID2 were detected by Western blot; (b) MGC-803 and BGC-823 cells were transfected with his-ID2 or flag-USP1, then exposed to CHX; the protein expression levels of his-ID2 and flag-USP1 were detected by Western blot at indicated time; (c) the interaction between USP1 and Ub was detected by Co-IP assay in MGC-803 and BGC-823 cells; (d) MGC-803 and BGC-823 cells were transfected with shUSP1 or flag-USP1, then exposed to PS-341; ubiquitinated ID2 was detected by Co-IP assay (^∗∗^*p* < 0.01; ^∗^*p* < 0.05).

As a deubiquitinase, USP1 can remove ubiquitin from target protein, so as to inhibit their degradation, whether USP1 stabilizing ID2 expression through its deubiquitination activity in GC was further explored. As shown in Figure 5(a), USP1 overexpression or knockdown increased or decreased ID2 expression, respectively, which was counteracted by proteasome inhibitor PS-341 (Figure 5(a)). Next, in order to detect the influences of USP1 on the rate of ID21degradation, MGC-803 and BGC-823 cells were transfected with his-ID2 and flag-USP1. As shown in Figure 5(b), USP1 overexpression significantly increased the half-life of ID2. The two experiments confirmed that USP1 stabilized ID2 expression. To test whether USP1 regulates the deubiquitination of ID2, Co-IP assay was firstly performed to define the interaction between endogenous USP1 and ID2. As shown in Figure 5(c), ID2 could be detected when USP1 was immunoprecipitated. Further experiments showed that USP1 knockdown or overexpression significantly increased or decreased the protein expression levels of ubiquitinated ID2, respectively (Figure 5(d)). Overall, these results demonstrate that USP1 stabilizes ID2 expression through deubiquitinating ID2 in GC.

## 4. Discussion

USP1 is a member of USPs which consists of 785 amino acids with a speculated molecular mass of 88.2 kDa [32]. As a member of USP, USP1 has been indicated important in regulating cancer proliferation and metastasis, such as in osteosarcoma and lung cancer [16, 20]. But the exact role of USP1 on GC metastasis and the underlying mechanism are still unclear. Our study is the first time to demonstrate that USP1 can promote GC metastasis by stabilizing ID2 expression via deubiquitinating ID2. Firstly, we examined the expression levels of USP1 in GC tissues and adjacent normal tissues and found that USP1 was overexpressed in the GC tissues, and relatively high-expression levels of USP1 conferred poor survival. Consistently, higher expression levels of USP1 were also found in all human GC cells compared with normal control, and USP1 knockdown significantly inhibited GC cell migration and distant liver metastasis. Thus, these results indicated that ID2 may function as an oncogene in GC.

ID2 is a member of four homologous proteins (Id1-Id4) which can dimerize with basic helix-loop-helix (bHLH) proteins and preventing them from binding DNA, so as to negatively regulate their biological functions, such as cell proliferation and differentiation [33, 34]. ID2 has been shown overexpressed in several cancer tissues or cells, such as salivary gland cancer and bladder cancer, and promote cancer invasion, proliferation, and metastasis [35–39]. In our study, consistent with the results in GC tissues [29], ID2 was also overexpressed in GC cells, and USP1 positively regulated ID2 expression. Moreover, we demonstrated that USP1 promoted GC metastasis by upregulating ID2 expression. And the mechanism by which USP1 regulated ID expression was further explored. Since Ub-proteasome pathway is an important degradation way for ID2, our results confirmed that the degradation of ID2 also depended on Ub-proteasome way in GC cells. As a deubiquitinase, USP1 has been shown to stabilize ID proteins in osteosarcoma cells [40–42]. Our study further confirmed that USP1 can stabilized ID2 expression through deubiquitinating ID2 in GC.

In conclusion, we firstly found that USP1 is overexpressed in GC tissues compared with adjacent normal tissues, and relatively high levels of USP1 conferred poor survival. Mechanically, we demonstrated that USP1 knockdown inhibits GC metastasis, and USP1 promoted GC metastasis via upregulating ID2 expression. And we further confirmed that USP1 stabilized ID2 expression through deubiquitinating ID2 in GC. Thus, our study provided a new biomarker therapy target for GC.

## Data Availability

The data used to support the findings of this study are included within the article.

## Supplementary Materials

Supplementary Materials Supplementary Table 1: primary sequences.
