# Supplementary material for: USP1 Promotes GC Metastasis via Stabilizing ID2
Source: Dis Markers. 2021 Nov 27;2021:3771990. doi: 10.1155/2021/3771990 (PMC8643267; doi:10.1155/2021/3771990)
Supplement: Supplementary Materials — Supplementary Table 1: primary sequences. [file 3771990.f1.docx]

| Supplementary Table 1. Primers sequences. | | |
| --- | --- | --- |
| name | sequences |  |
| **Primers for real-time PCR:** |  |  |
| USP1 Forward Primer | 5'-ATGCCTGGTGTCATACCTAGT-3′ |  |
| USP1 Reverse Primer | 5'-CAGTCCCACAAATGGTAACAAGT-3′ |  |
| ID2 Forward Primer | 5'-CTGCGCCAACCTCGTAAGG-3′ |  |
| ID2 Reverse Primer | 5'-TTCCTCATCACAGTTGCTCCC-3′ |  |
| GAPDH Forward Primer | 5'-TGTGGGCATCAATGGATTTGG-3′ |  |
| GAPDH Reverse Primer | 5'-ACACCATGTATTCCGGGTCAAT-3′ |  |
| **Primers for plasmids construct:** |  |  |
| pCMV-flag-USP1 sense | 5’-CGCGGATCCGCTCCCAATGCTTCCTGCCT-3’ |  |
| pCMV-flag-USP1 antisense | 5’-CCGGAATTCGGTCACCCTCCAATACAATAAG-3’ |  |
| pCMV-his-ID2 sense | 5’-ACGGGCCCTCTAGACTCGAGCGCCACCATGGACTACAAGGATGACG-3’ |  |
| pCMV-his-ID2 antisense | 5’-TTAAACTTAAGCTTGGTACCTCAAATACAATAACATGCCAGG-3’ |  |
| pCMV-HA-Ub sense | 5’-GAGGATCCCCGGGTACCGGTCGCCACCATGAGGTGGTTCCTGCCCTGGAC-3’ |  |
| pCMV-HA-Ub antisense | 5’-CACACATTCCACAGGCTAGCTAAGCATAGTCTGGGACATCATAAGGGTAGTTGGCAATTTCTGAGAAGTC-3’ |  |
| **The target sites of shRNA:** |  |  |
| shUSP1-#1 | GGCAGATTACGGCATCAGA |  |
| shUSP1-#2 | GGGATTTAATGACCTTTGA |  |
| shID2-#1 | GGAGAAGCCTCTCATCTTA |  |
| shID2-#2 | GCTCAGTGGCACAAGTGAA |  |
